# Deep learning-based method to accurately estimate breast tissue optical properties in the presence of the chest wall

**DOI:** 10.1117/1.JBO.26.10.106004

**Published:** 2021-10-20

**Authors:** Menghao Zhang, Shuying Li, Yun Zou, Quing Zhu

**Affiliations:** a Washington University in St. Louis, Department of Electrical and Systems Engineering, St. Louis, Missouri, United States; b Washington University in St. Louis, Department of Biomedical Engineering, St. Louis, Missouri, United States; c Washington University School of Medicine, Department of Radiology, St. Louis, Missouri, United States

**Keywords:** diffuse optical tomography, deep learning, breast tissue optical properties

## Abstract

**Significance:** In general, image reconstruction methods used in diffuse optical tomography (DOT) are based on diffusion approximation, and they consider the breast tissue as a homogenous, semi-infinite medium. However, the semi-infinite medium assumption used in DOT reconstruction is not valid when the chest wall is underneath the breast tissue.

**Aim:** We aim to reduce the chest wall’s effect on the estimated average optical properties of breast tissue and obtain accurate forward model for DOT reconstruction.

**Approach:** We propose a deep learning-based neural network approach where a convolution neural network (CNN) is trained to simultaneously obtain accurate optical property values for both the breast tissue and the chest wall.

**Results:** The CNN model shows great promise in reducing errors in estimating the optical properties of the breast tissue in the presence of a shallow chest wall. For patient data, the CNN model predicted the breast tissue optical absorption coefficient, which was independent of chest wall depth.

**Conclusions:** Our proposed method can be readily used in DOT and diffuse spectroscopy measurements to improve the accuracy of estimated tissue optical properties.

## 1 Introduction

Near-infrared (NIR) diffuse optical tomography (DOT) is a non-invasive functional imaging technique that delivers light into breast tissue and uses image reconstruction techniques to recover the hemoglobin concentrations of breast lesions, which are directly related to tumor angiogenesis.^1^^–^^4^ In diffuse reflection measurements, patients are typically scanned in a supine position, and several sets of optical measurements are simultaneously made from the lesion location and a contra-lateral region of the normal breast.^5^ The normalized perturbation between the lesion and reference measurements is used for imaging reconstruction. To ease the ill-posed, ill-conditioned DOT reconstruction problem, other modalities, such as ultrasound (US),^6^ MRI,^7^ and x-ray CT,^8^ have been introduced to provide prior information, such as the lesion’s location and size. US-guided DOT has been applied clinically in several studies because of its low cost and easy implementation.^9^^–^^11^

DOT reconstruction requires two steps to recover lesion optical properties. The first step involves computation of a weight matrix to establish a forward model of the light propagation inside the diffusive medium, using average tissue optical properties estimated from optical measurements collected from the normal breast tissue.^12^ In the second step of DOT reconstruction, the weight matrix computed from the average tissue optical properties is used to relate the unknown lesion optical properties to the perturbation measurements, which are the differential measurements from the lesion side or target side and healthy breast or reference side. Iterative optimization algorithms, such as nonlinear iterative gradient-based optimization methods,^13^ are typically used to reconstruct lesion optical properties. Thus, accurately estimating the average optical properties of the normal breast tissue is the first important step in DOT reconstruction. This paper is focused on a new neural network approach to accurately estimating the average optical properties of the normal breast tissue.

The most common estimation method of normal breast tissue, using frequency-domain system measurements, is based on the slopes of two plots: the least-squares fitted log amplitude versus the source-detector distance, and the phase versus source-detector distance.^14^^,^^15^ Although this method is easy to implement and relatively robust to measurement errors, it assumes that the breast tissue is a homogenous, semi-infinite medium, and this assumption is not valid when the chest wall is at a shallow depth, <2.5  cm below the skin surface.^16^^,^^17^ In this case, a more accurate approach to estimate the optical properties is required. Earlier, our group developed a two-layer model which used either a two-layer analytical solution of the diffuse equation or the finite-element method (FEM) combined with nonlinear optimization methods to iteratively estimate the breast tissue and chest wall optical properties for constructing more accurate forward models. However, the nonlinear optimization methods were time consuming, and the fitted optical properties depended highly on the initial estimates.^18^^,^^19^

In recent years, deep neural networks have achieved remarkable success in various medical imaging applications.^20^ Deep learning has been applied to DOT image reconstruction,^21^^–^^23^ where the neural network is trained to learn the nonlinear relationship between the optical anomalies and the photon scattering physics to acquire better DOT image quality as compared with the traditional reconstruction algorithm. Deep learning has also been applied to spatial frequency-domain imaging (SFDI) to recover the optical properties accurately and quickly from the diffuse reflectance image.^24^^–^^27^ Additionally, Sabir et al.^28^ used a popular deep learning algorithm, convolutional neural network (CNN), to estimate the bulk tissue optical properties. However, they studied only a homogenous, semi-infinite medium and did not consider the presence of the chest wall.

Here, we propose a deep learning-based approach in which a neural network model is trained to simultaneously estimate the optical properties of both the breast tissue and the chest wall from measurements of normal breast tissue. The chest-wall depth is estimated from co-registered US images. Simulation data are used to train the proposed model, and phantom experimental data are used to improve its accuracy. Test results from simulation, phantom, and clinical data show that the proposed model estimates the optical properties of the breast tissue in the presence of the chest wall more accurately than the FEM-based two-layer fitting algorithm and the traditional slope-based fitting algorithm, aiding the construction of more accurate forward models in DOT reconstruction. To the best of our knowledge, this is the first time that a deep learning neural network model has been used to estimate the bulk optical properties of breast tissue and the underlying chest-wall.

## 2 Methods

### 2.1 Photon Migration in a Diffusive Medium and DOT Reconstruction

Photon migration in a diffusive medium, such as breast tissue, can be modeled using the radiative transfer equation (RTE), but its solution is known to be computationally challenging. Therefore, the diffusion equation, a low-order approximation of the RTE, is used to generate approximated solutions.^12^ The frequency-domain diffusion equation is shown in Eq. (1):

$$(\mu_{a}(\overset{\to }{r})+\frac{i\omega }{c_{m}(\overset{\to }{r})})\Phi (\overset{\to }{r},\omega )-\nabla \cdot [D(\overset{\to }{r})\nabla \Phi (\overset{\to }{r},\omega )]=S(\overset{\to }{r},\omega ),$$ (1)

where $\mu_{a}$ and D denote the absorption and the diffusion coefficients, respectively, $c_{m}$ represents the speed of light inside the medium, $S(\overset{\to }{r},t)$ represents the source term, and $\Phi (\vec{r},t)$ is the generated photon density fluence rate at position $\vec{r}$ with modulation frequency ω.

The forward model used in DOT reconstruction, with measurements collected from a reflection geometry, is computed from the analytic solution of the diffusion equation based on a homogeneous semi-infinite medium. The optical properties of healthy tissue are assumed to be similar between the two breasts, thus, the average optical properties are estimated from measurements obtained from healthy breast tissue (see Sec. 2.3) and used to compute a weight matrix, W. However, a more accurate W can be computed from a two-layer medium including the chest wall underneath the breast tissue. Hence, obtaining accurate optical properties of the average breast tissue and chest wall is an essential first step in building an accurate forward model in DOT reconstruction.

### 2.2 US-Guided DOT System and Data Calibration

**Fig. 1:**
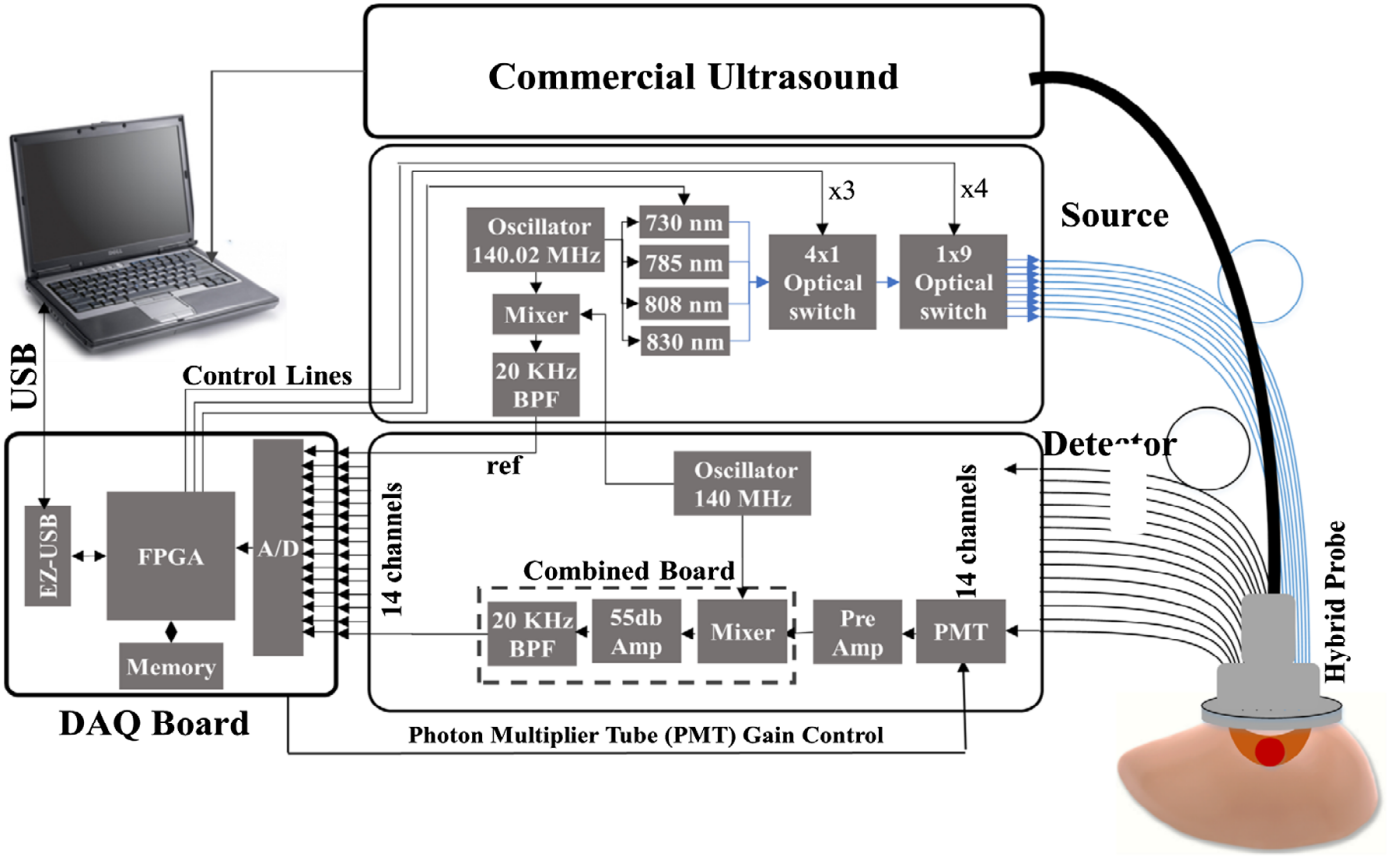
Block diagram of the US-guided DOT system. The system contains 9 sources and 14 detectors with an US transducer incorporated in the center of the probe.^30^

We have developed a compact, frequency domain, US-guided DOT system with a handheld probe (Fig. 1), and it was used here for phantom and patient experiments. The DOT system uses four laser diodes with wavelengths of 730, 785, 808, and 830 nm, and it incorporates 14 parallel photomultiplier (PMT) detectors and a commercial US system. Nine illumination source fibers are placed on one side of the US transducer, and detectors are placed on the other side, with source-detector separations varying from 3 to 7.6 cm. Each laser diode is modulated at 140 MHz and delivers light sequentially to the nine source locations on the handheld probe. The output of each detection channel is demodulated to 20 kHz and further amplified and filtered before passing to an analog-to-digital converter (ADC).^29^

Since individual PMTs have different gains and individual laser diodes have different powers, we calibrate the source powers and detector gains for all detection channels and source positions.^29^ A set of measurements obtained from a homogenous intralipid solution with known background absorption and scattering properties is used to calculate the gains associated with detectors and sources, based on the least-squares method. These calibration parameters are applied to later measurements acquired from the phantom or patient.

### 2.3 Slope-Based Fitting Algorithm

The most common algorithm used to compute the average absorption and reduced scattering coefficients from frequency-domain reflection measurements is based on slopes of log amplitude versus source-detector distance, ρ, and phase versus ρ. In this algorithm, the medium is treated as homogenous and semi-infinite. Under this condition, when the source-detector separations are larger than the transport mean free path, which is usually a couple of millimeters, the calibration amplitude $\log(A_{r}\rho^{2})$ and phase $\phi_{r}$ have linear relationships with ρ.^15^ The two slopes $k_{i}$ and $k_{r}$ represent the imaginary and real parts of the wavenumber k in diffusion theory. The absorption coefficient, $\mu_{a}$, and reduced scattering coefficients, $\mu_{s}^{\prime }$, can be computed as

$$\mu_{a}=\frac{\omega }{c_{m}}{\left[\tan(2{\;\tan}^{-1}\;\frac{k_{r}}{k_{i}})\right]}^{-1},$$ (2)

$$\mu_{s}^{\prime }=\frac{k_{r}^{2}+k_{i}^{2}}{3\sqrt{\mu_{a}+{\left(\omega /c_{m}\right)}^{2}}},$$ (3)

where ω is the modulation frequency and $c_{m}$ represents the speed of light inside the medium. However, the semi-infinite assumption is not valid in the presence of the shallow chest wall, which significantly increases the fitted error from the slope-based fitting algorithm.

### 2.4 Two-Layer FEM Model Based Fitting Approach

To acquire the optical properties with the presence of the chest wall, our group has developed a two-layer model that used an FEM combined with a nonlinear optimization method to estimate the optical properties of the breast tissue and the chest wall.^30^ The FEM was used to generate the forward measurements from a 3D two-layer mesh, where the depth and angle of the interface of the breast tissue and the chest wall were determined by co-registered US images. To solve the nonlinear problem between optical properties and the optical measurements given as

$$\varphi (r,\omega )=f(\mu_{a},\;\mu_{s}^{\prime },\mu_{a\_\text{chest}},\mu_{s\_\text{chest}}^{\prime }),$$ (4)

where $\mu_{a}$, $\mu_{s}^{\prime }$, $\mu_{a\_\text{chest}}$, $\mu_{s\_\text{chest}}^{\prime }$ are the four optical properties we aim to recover for both the breast tissue and the chest wall. A nonlinear regression algorithm based on the Nelder–Mead method is applied to estimate these four parameters. This algorithm is chosen based on its simplicity and good convergence. However, it is slow on convergence and requires 150 to 230 iterations.^31^ We refer to this method as a two-layer fitting algorithm.

### 2.5 Deep Learning-Based Approach

The problem can be formulated as a nonlinear function,

$$\mu_{a},\mu_{s}^{\prime },\mu_{a\_\text{chest}},\mu_{s\_\text{chest}}^{\prime }=f(\theta ,C,O),$$ (5)

where $\mu_{a}$, $\mu_{s}^{\prime }$, $\mu_{a\_\text{chest}}$, $\mu_{s\_\text{chest}}^{\prime }$ are the four optical properties to be estimated, θ is the neural network, C is the chest wall vector, which is not considered in the slope-based fitting algorithm, and O is the optical measurements collected by the DOT system. The neural network with parameters θ estimates the four optical properties by learning the nonlinear mapping between the optical measurements and the average tissue optical properties.

#### 2.5.1 Neural network structure

Here we propose a deep learning-based approach to learn the nonlinear mapping between the measurements and the optical properties of the breast tissue and the chest wall. This approach is accomplished by performing supervised training on a CNN model. CNN is a popular neural network that uses at least one convolutional layer in its architecture.^32^

**Fig. 2:**
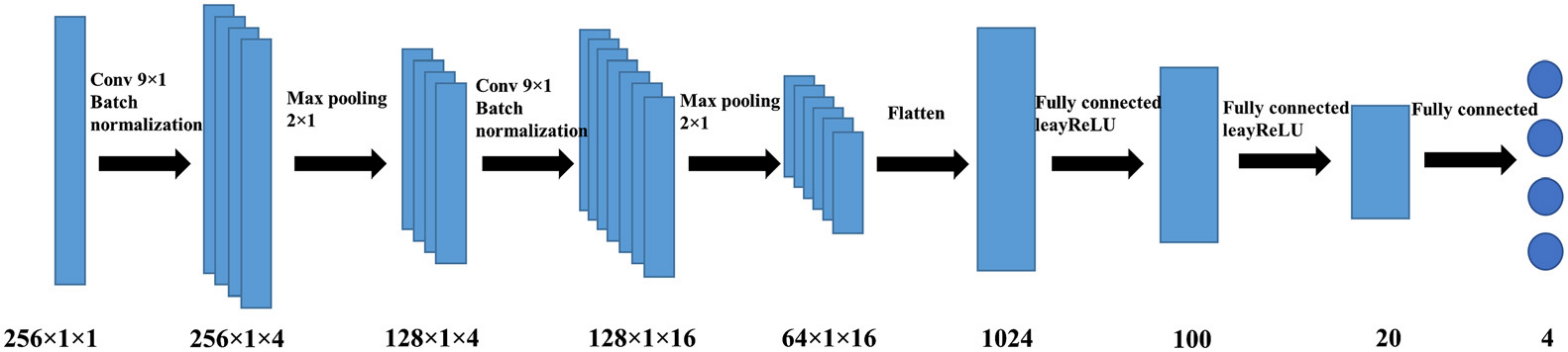
Neural network architecture for obtaining the optical properties of both breast tissue and the chest wall. Two convolutional layers with a kernel size of (9,1) are followed by a batch normalization layer and a max-pooling layer with a kernel size of (2,1). Two fully connected layers learn the mapping between the features extracted by the convolution layers and the optical properties of both the breast tissue and the chest wall.

As shown in Fig. 2, the CNN model uses optical measurements collected from our DOT system and chest wall depth values from the co-registered US images as inputs, and from these, it predicts the absorption coefficients, $\mu_{a}$, and the reduced scattering coefficients, $\mu_{s}^{\prime }$, for both the breast tissue and the chest wall. The measurements and the depths of the chest wall are aligned in a vector form to feed into the neural network. The 256×1×1 input consists of 126 elements of the log amplitude, 126 elements of the phase, and four elements of the chest wall depths, which are measured at four equally spaced locations from left to right in the US image. The architecture of our network consists of two convolutional layers with kernel size (9,1) and zero padding, each followed by a batch-normalization layer and a max-pooling layer with a pool size of (2,1), and two fully connected layers. Batch normalization is a method used to accelerate and stabilize the neural network by mitigating the problem of internal covariate shift.^33^ The convolutional layers extract the features from the input data, and the fully connected layers learn the nonlinear mapping between features and the optical properties we aimed to recover. To handle the negative values, which obtained from the optical measurements and the preprocessing of the data, inside the neural network the activation function is chosen as leakyReLU, an adaptation of the ReLU function. The ReLU activation function is defined as the positive part of its input argument, thus it sets all negative values to zero.^34^ Due to the vanishing gradient problem in the ReLu, the leakyReLU is introduced to mitigate the problem.

#### 2.5.2 Training and testing datasets

##### Simulation data

Forward measurements were generated using the FEM. The simulation geometry was set as a two-layer model, with homogenous breast tissue on the top and the homogenous chest wall on the bottom.^18^ The data were generated based on six different parameters: the tissue absorption coefficient ($\mu_{a}$), tissue reduced scattering coefficient ($\mu_{s}^{\prime }$), chest wall absorption coefficient ($\mu_{a\_\text{chest}}$), chest wall reduced scattering coefficient (${\mu^{\prime }}_{s\_\text{chest}}$), chest wall depth, and the chest wall tilt angle. A total of 15,744 sets of measurements were generated, and 90% of the measurements were randomly chosen as training sets. The values of the optical properties of the breast tissue and chest wall were chosen based on Refs. 35 and 36. In many clinical cases, the deep chest wall does not have a significant effect on the optical measurements, and the background breast tissue can be modeled as a homogenous and semi-infinite medium. However, when the chest wall is located 2.5 cm or less below the skin surface, its effect on the measurements cannot be neglected, based on the simulation results. Accordingly, the range of chest wall depths was set between 1.5 and 3 cm. A 1% Gaussian noise was added into the simulation data to mimic DOT system noise (Table 1).

**Table 1:** Simulation parameters.

Parameters, units	$\mu_{a}$, $\;{\mathrm{cm}}^{-1}$	$\mu_{s}^{\prime }$, $\;{\mathrm{cm}}^{-1}$	$\mu_{a\_\text{chest}}$, $\;{\mathrm{cm}}^{-1}$	$\mu_{s\_\text{chest}}^{\prime }$, $\;{\mathrm{cm}}^{-1}$	Chest wall depth, cm	Chest wall angle, deg
Range	0.01–0.1	4–10	0.1–0.24	4–10	1.5–3	-15–+15

##### Digital breast phantom data

**Fig. 3:**
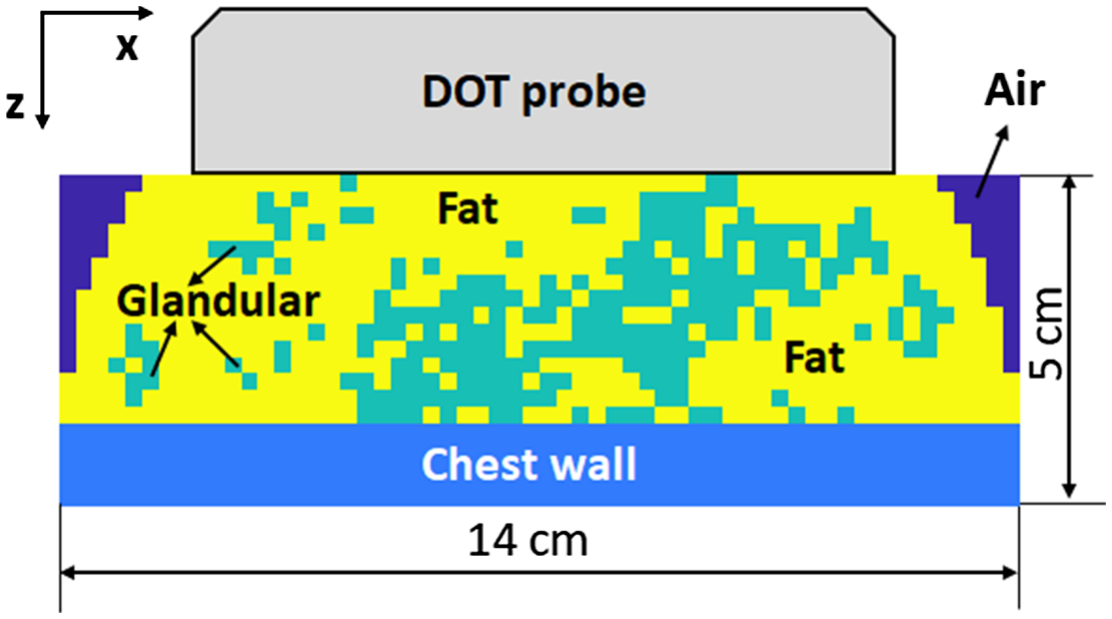
Cross-section of the digital breast phantom compressed by the hand-held DOT probe. The digital breast has a size of 14 cm×14 cm by 5 cm with a voxel size of 0.5  mm×0.5  mm×0.5  mm.

To validate the proposed model, we utilized the VICTRE digital breast phantom^37^ that mimicked a realistic human breast with a heterogeneous tissue structure. The digital breast measured 14 cm in the x and y direction and 8 cm in the z direction and had a voxel size of $0.5\times 0.5\times 0.5\;{\mathrm{mm}}^{3}$. Here, we set the digital breast phantom to consist of both fat and fibroglandular tissues with different optical properties, placed randomly inside the breast to mimic the heterogeneity of breast tissue. We set the optical properties of the fat tissue as $\mu_{a}=0.02{\;\mathrm{cm}}^{-1}$ and $\mu_{s}^{\prime }=5\;{\mathrm{cm}}^{-1}$; for fibroglandular tissue, they were set as $\mu_{a}=0.04\;{\mathrm{cm}}^{-1}$ and $\mu_{s}^{\prime }=8\;{\mathrm{cm}}^{-1}$. The fraction of fibroglandular tissue ranged from 20% to 80%, with the rest of breast tissue are set to fat tissue. The chest wall was placed between 1.5 cm and 2.5 cm beneath the tissue surface. Then, the digital breast was numerically compressed to 5 cm using nearest neighbor interpolation to simulate the breast compressed by the hand-held DOT probe. Then the digital phantom was down sampled to a voxel size of $2.5\times 2.5\times 2.5\;{\mathrm{mm}}^{3}$ for the Monte Carlo simulations. In all, 24 sets of digital breast phantom data were generated. The ground truth optical properties for the breast tissue in the heterogenous scenario were computed as the weighted averages of the $\mu_{a}\;$ and $\mu_{s}^{\prime }$ values for fat tissue and fibroglandular tissue (Fig. 3).

##### Phantom experiment data

**Fig. 4:**
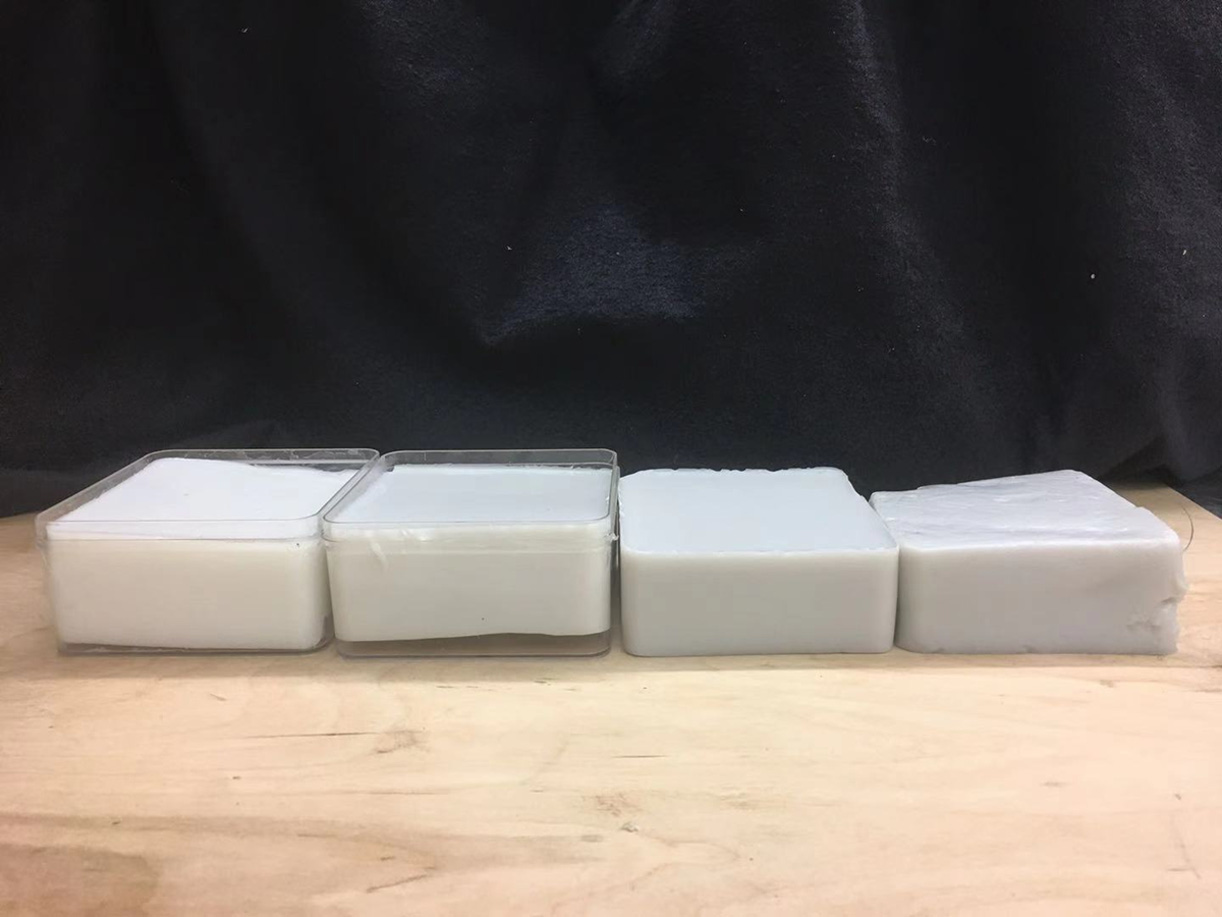
Four high contrast solid phantoms for mimicking the chest wall with the size of 10 cm by 10 cm by 4 cm. Their absorption coefficients range from 0.095 to 0.18, and their reduced scattering coefficients range from 5.6 to $7.1\;{\mathrm{cm}}^{-1}$.

To mimic breast tissue in phantom experiments, 5% aqueous intralipid solutions were mixed with different amounts of black ink to provide absorption coefficients ranging from 0.02 to $0.06\;{\mathrm{cm}}^{-1}$ and reduced scattering coefficients ranging from 5.8 to $6.7\;{\mathrm{cm}}^{-1}$, values within the range of our simulation dataset. Solid high optical contrast phantoms were made of intralipid^®^ 20% IV fat emulsion (2B6022, Baxter International Inc., Deerfield, Illinois), 5% Type A 300 Bloom gelatin derived from acid-cured porcine skin (G2500, Sigma-Aldrich Corp., St. Louis, Missouri),^38^ and black ink, which was used to increase the absorption coefficients of the phantoms. Four solid phantoms, shown in Fig. 4, were made to mimic the chest wall, with absorption coefficients varying from 0.095 to $0.18\;{\mathrm{cm}}^{-1}$ and reduced scattering coefficients varying from 5.6 to $7.1\;{\mathrm{cm}}^{-1}$, values which again were within the range of the simulation dataset. The high contrast chest wall mimicking phantoms were submerged in the liquid intralipid solutions at 1.5 to 2.5 cm below the probe surface in the liquid. The handheld probe was rotated to mimic different chest wall angles. The chest wall depths and angles were obtained from co-registered US images. In total, 784 sets of measurements were collected: 70% of them were used in fine-tuning the proposed model, while the other 30% were used in testing.

##### Patient data

Our US-guided DOT technology has translated to patient studies.^9^ The protocol for this study was approved by the local Institutional Review Boards and was compliant with the Health Insurance Portability and Accountability Act. All patients signed the informed consent form, and all patient data used in this study were deidentified. A total of 12 patients with shallow chest walls (<2.5  cm below the skin surface) was studied to evaluate the performance of the CNN model.

#### 2.5.3 Implementation details

The CNN model was trained on the simulation data and fine-tuned with part of the phantom data. Before training, all training data was normalized to have zero mean and unit standard deviation. The testing data was also normalized based on mean and standard deviation of the training data. Due to the signal-to-noise ratio consideration, all measurements from source-detector separations >7  cm were removed. We also removed data outliers at a large distance from the fitted line obtained from the slope-based fitting algorithm. The loss function was defined as the mean square error of all four output values. The optimization scheme was the Adam optimizer, an extension to stochastic gradient descent that has recently seen broader adoption in deep learning applications. The Adam optimizer can be viewed as a combination of RMSprop^39^ and stochastic gradient descent with momentum. It has the best overall performance across a variety of tasks.^40^ The learning rate was set to 0.001, with a momentum of 0.9. A learning rate decay of 0.1 after every 100 epochs was applied to help the neural network converge and avoid oscillation.^41^ We use a batch size of 32 and trained the model for 200 epochs. An early-stopping criterion based on the validation loss after every five epochs is applied and 10-fold validation during the training is used. After training, the model was fine-tuned by the phantom experiment data, with a learning rate of 0.0001 for 100 epochs.

### 2.6 Evaluation of the Performance of the CNN Model

To quantify the performance of the CNN model, relative error was introduced in simulation and phantom studies:

$$\text{relative error}=\frac{abs|\text{prediction}-\text{ground truth}|}{\text{ground truth}},$$ (6)

where the ground truth is the known optical properties of the breast tissue and the chest wall, and the prediction is the output of the model. The absolute error is normalized by the ground truth to provide the relative error.

## 3 Results

### 3.1 Simulation Results

**Fig. 5:**
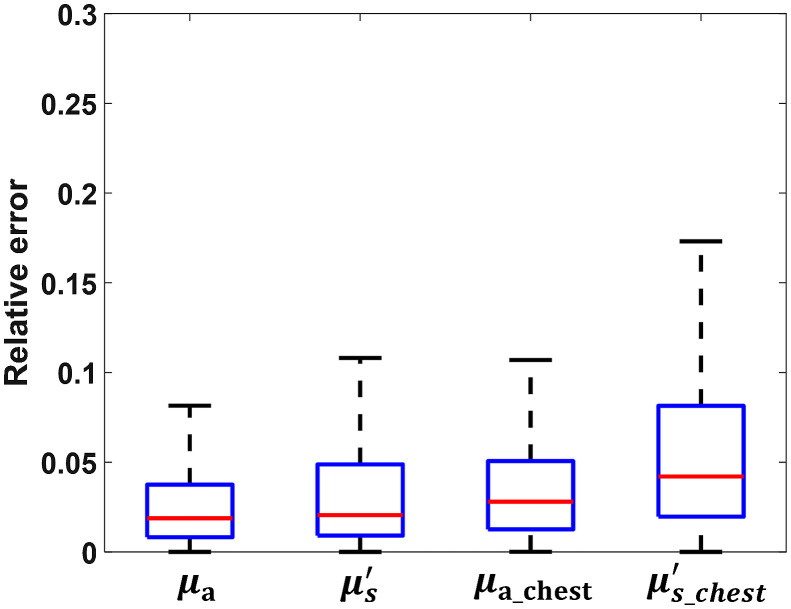
Relative error of the testing results on simulation data. $\mu_{a}$ and $\mu_{s}^{\prime }$ are the absorption coefficient and reduced scattering coefficient of the breast tissue layer; $\mu_{a\_\text{chest}}$ and $\mu_{s\_\text{chest}}^{\prime }$ are the absorption coefficient and reduced scattering coefficient of the chest wall layer.

Among the 15,744 sets of measurements, 10% were directly tested on the CNN model. Figure 5 shows the testing results for all four optical properties. $\mu_{a}$ has a mean relative error of 3.25%, the best testing result among all four output parameters. $\mu_{s\_\text{chest}}^{\prime }$ has the worst performance of all four, with a mean relative error of 6.26%. The overall performance of the CNN model in the breast tissue is better than the performance in the chest wall. This difference is expected because most photons are absorbed or reflected before they reach the chest wall, and the collected measurements have less information about the chest wall than the breast tissue.

**Fig. 6:**
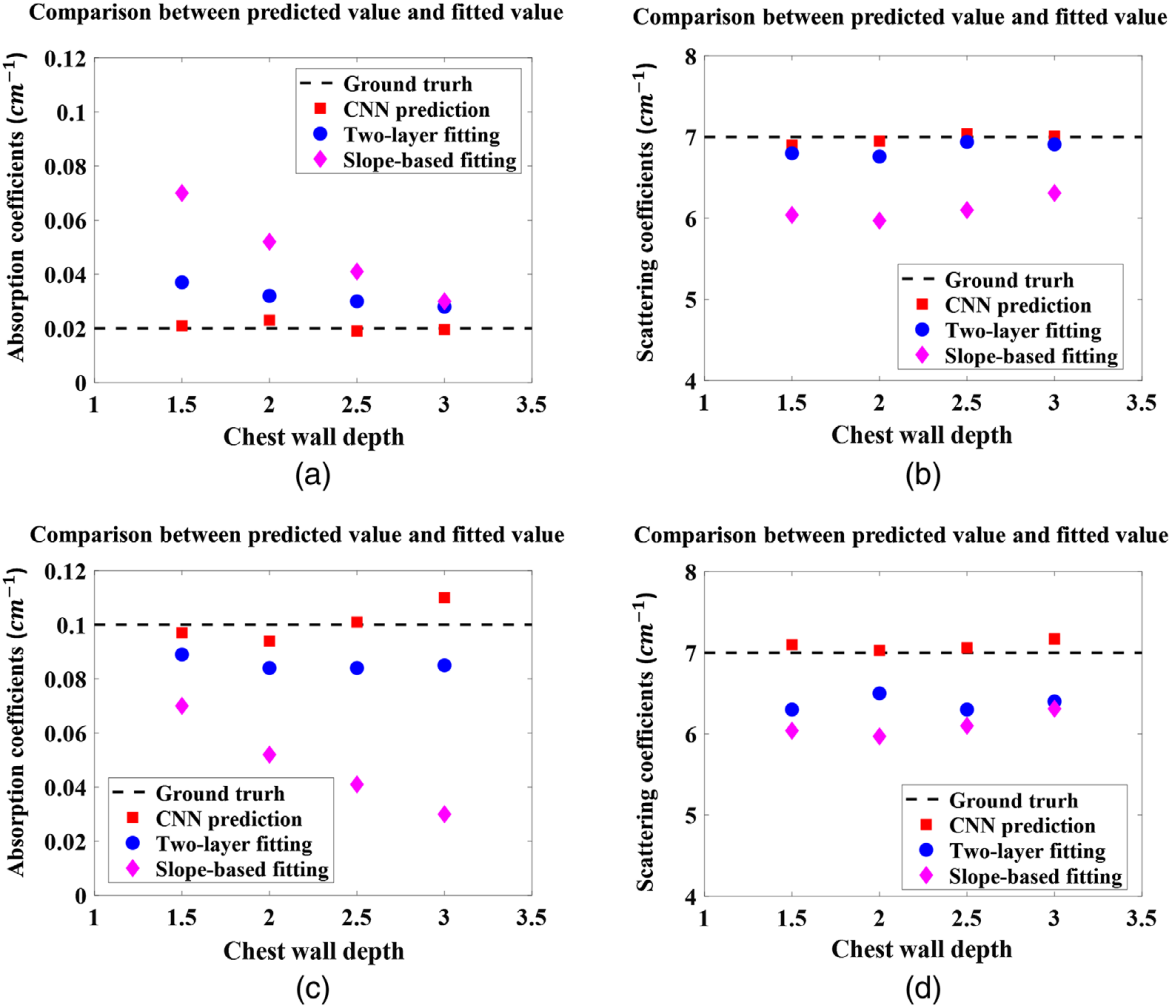
The absorption coefficients of (a) the breast tissue layer and (c) the chest wall layer, and the reduced scattering coefficients of (b) the breast tissue layer and (d) the chest wall layer, based on the CNN prediction, two-layer fitting algorithm, and the slope-based fitting, with the dashed lines indicating the ground truth values.

To compare the performance of the CNN model with the traditional slope-based fitting algorithm and the two-layer fitting algorithm, four sets of simulated measurements were generated, which appear in neither the training nor the testing datasets but have optical properties with values in the range of the training data. These four additional sets of data were tested on the CNN model and fitted by the two-layer fitting algorithm and slope-based fitting algorithm. The ground truth values are $\mu_{a}=0.02\;{\mathrm{cm}}^{-1}$ and $\mu_{s}^{\prime }=7\;{\mathrm{cm}}^{-1}$, $\mu_{a\_\text{chest}}=0.12\;{\mathrm{cm}}^{-1}$ and $\mu_{s\_\text{chest}}^{\prime }=7\;{\mathrm{cm}}^{-1}$. The chest wall was placed at 1.5 to 3 cm depths, with a step size 0.5 cm, and was not tilted. For comparison, the predicted results from the CNN model and the fitted results from the two-layer fitting algorithm and the slope-based algorithm are shown in Fig. 6, where the depth of the chest wall is presented on the x axis. Because the slope-based fitting algorithm assumes the medium is homogenous and semi-infinite, it calculates $\mu_{a}$ and $\mu_{s}^{\prime }$ for the breast tissue only, not for both the breast tissue and the chest wall, so we use these two values to represent the chest wall as well. The results show that the error in the value of $\mu_{a}$ in the breast tissue obtained by slope-based algorithm decreases when the chest wall is deeper, and the error in $\mu_{a}$ in the chest wall layer increases when the chest wall is deeper. These opposing trends reflect the fact that when the chest wall is closer to the skin surface, the measurements are more affected by the higher absorption coefficients of the chest wall. The errors in $\mu_{s}^{\prime }$ are relative consistent because the $\mu_{s}^{\prime }$ values for both breast tissue and the chest wall were set to $7\;{\mathrm{cm}}^{-1}$. Both the CNN and two-layer fitting algorithm provide accurate results; however, the CNN has the best accuracy among the three approaches. Using the two-layer fitting algorithm, the average running time for one set of measurements is 36 min, too long to be useful in clinical studies as compared with the several seconds for slope-based fitting and 20 s for CNN prediction. Among the three methods, the CNN model best estimates the optical properties of both the breast tissue and the chest wall accurately and consistently, despite varying chest wall depths.

### 3.2 Digital Breast Phantom Results

**Fig. 7:**
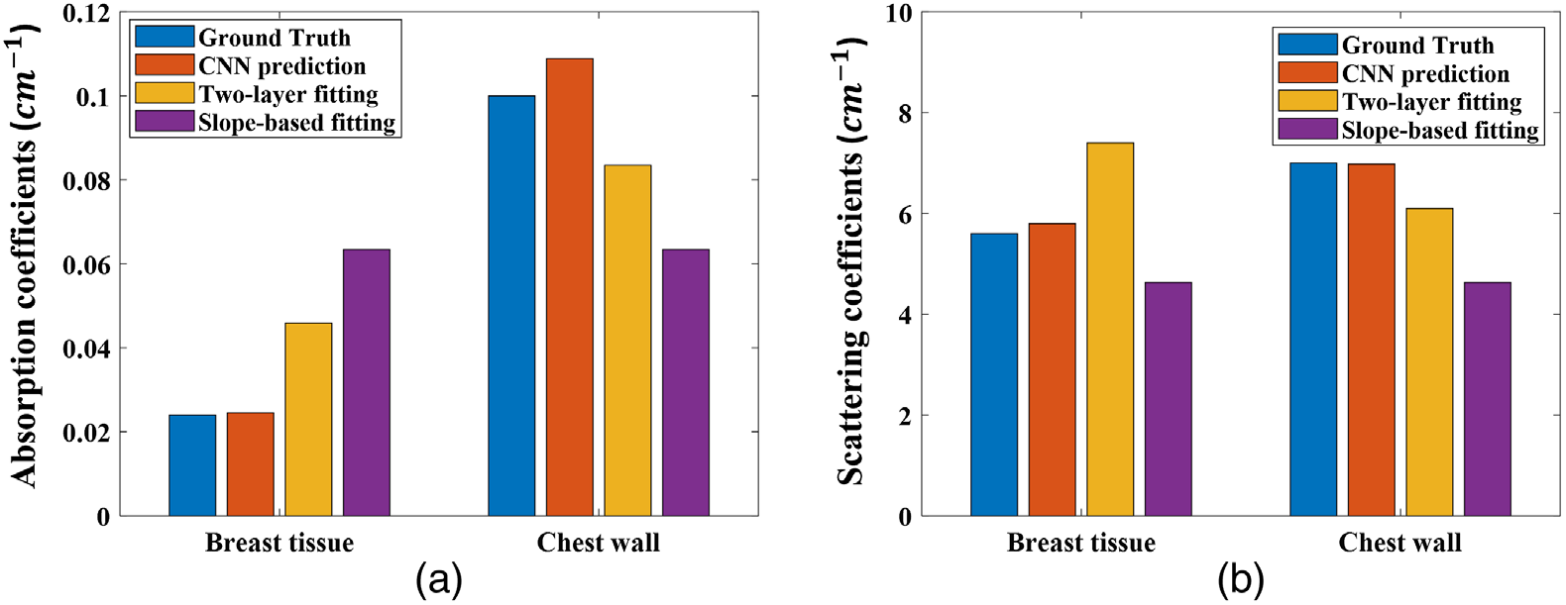
Comparison of the estimated optical properties of both breast tissue and the chest wall of the digital breast phantom.

The digital breast phantom data was directly tested on the trained model, and an example of the results is shown in Fig. 7. Although the neural network is trained on homogenous breast tissue data and fine-tuned on a similar scenario, it can still accurately predict the optical properties of heterogenous breast tissue. As a broader generalization and as mentioned above, we also observed that as the chest wall depth becomes shallower, the errors of the slope-based fitting algorithm increase. The two-layer fitting algorithm has more accurate results than slope-based fitting, but is still worse than the CNN model, which has the highest accuracy among all three methods. Across all 24 sets of digital breast phantom data, the CNN predictions have an 6.64% mean relative error on $\mu_{a}$, an 8.52% mean relative error on $\mu_{s}^{\prime }$, an 8.34% mean relative error on $\mu_{a\_\text{chest}}$, and an 11.89% mean relative error on ${\mu^{\prime }}_{s\_\text{chest}}$.

### 3.3 Phantom Experiment Results

**Fig. 8:**
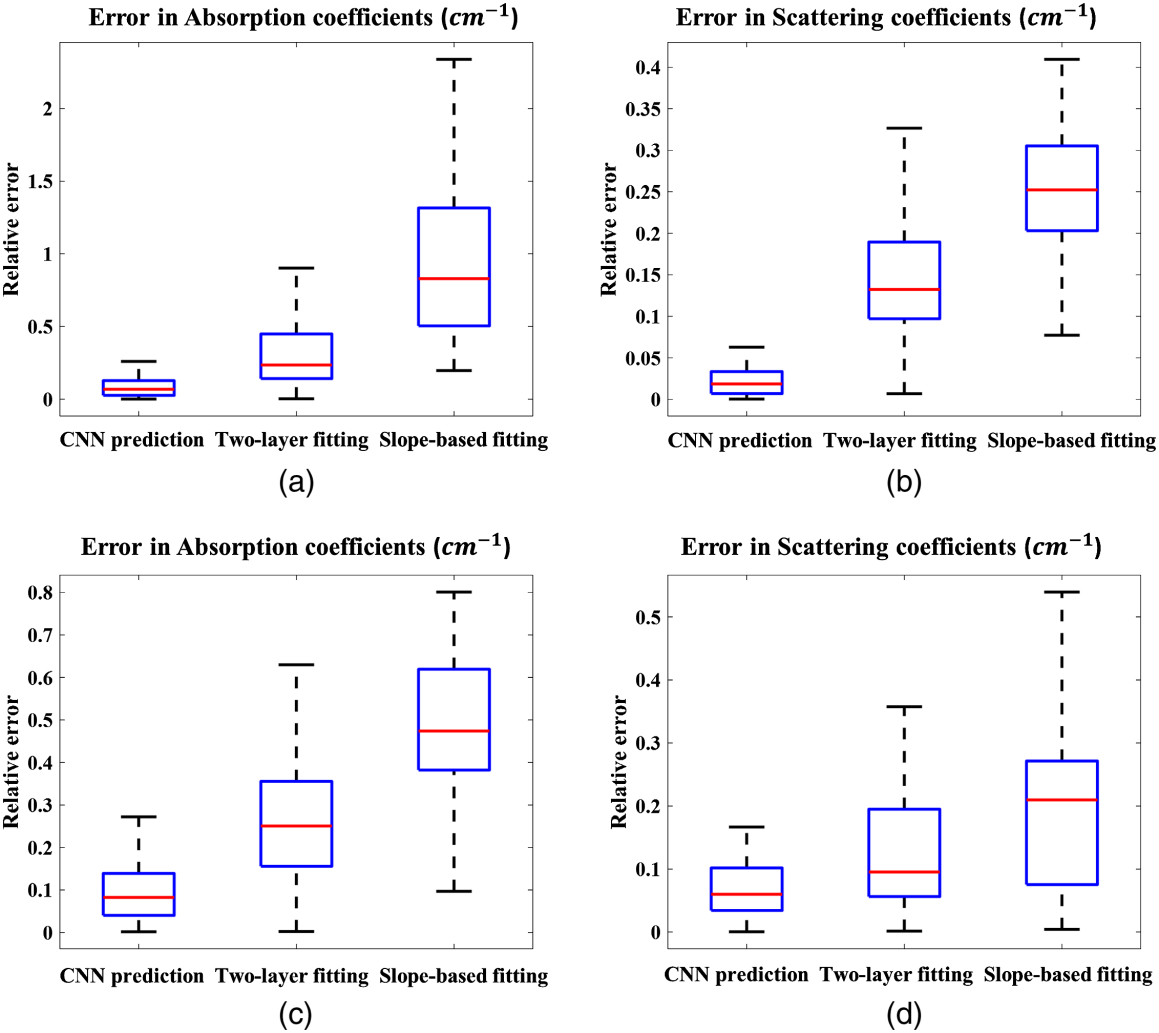
Relative errors of the neural network predictions, two-layer fitting and slope-based fittings. (a), (b) Relative errors of the absorption coefficient, $\mu_{a}$, and reduced scattering coefficient, $\mu_{s}^{\prime }$, for the breast tissue. (c), (d) Relative errors of the absorption coefficient and reduced scattering coefficient of the chest wall.

The phantom experiments described in Sec. 2.5.2 were conducted to fine-tune the CNN model and further evaluate its performance. For comparison, Fig. 8 shows the predictions of the CNN and the fitted values of both the two-layer fitting and the slope-based algorithm. The CNN model shows promising results in reducing the relative estimation error in the optical properties of the breast tissue and the chest wall, especially in the absorption coefficients of the breast tissue. Notably, when the chest wall is shallow, the slope-based fitted values approximate the average of the breast tissue and the chest wall values, leading to an extremely high relative error in $\mu_{a}$ for the breast tissue. Two-layer fitting performs better than slope-based fitting but is still worse than CNN prediction based on the relative error metric. In the two-layer fitting, we also observe larger errors in the optical properties of the chest wall when the chest wall is deeper than 2 cm. The CNN model predicts accurate values despite the change in the chest wall depth.

To summarize, Table 2 shows the mean relative errors across digital breast phantom data, and phantom experimental data using CNN, two-layer fitting, and slope-based fitting. The CNN model shows great promise in reducing errors in estimating the optical properties of the breast tissue in the presence of a shallow chest wall. Compared with slope-based fitting algorithm, the errors of breast tissue $\mu_{a}$ are reduced by 61% for digital breast phantom data, and 83% for phantom experiment. Compared with two-layer fitting algorithm, the corresponding errors are reduced by 35%, and 28%, respectively.

**Table 2:** Mean relative errors of digital breast phantom and phantom experimental data.

		$\mu_{a}$ (%)	$\mu_{s}^{\prime }$ (%)	$\mu_{a\_\text{chest}}$ (%)	$\mu_{s\_\text{chest}}^{\prime }$ (%)
Digital breast phantom	CNN prediction	6.64	8.52	8.34	11.89
	Two-layer fitting	41.23	19.81	20.45	35.72
	Slope-based fitting	67.54	24.34	44.51	77.34
Phantom	CNN prediction	6.55	4.76	8.38	7.94
	Two-layer fitting	34.99	14.11	28.87	12.60
	Slope-based fitting	89.65	24.34	44.13	19.78

### 3.4 Clinical Examples

**Fig. 9:**
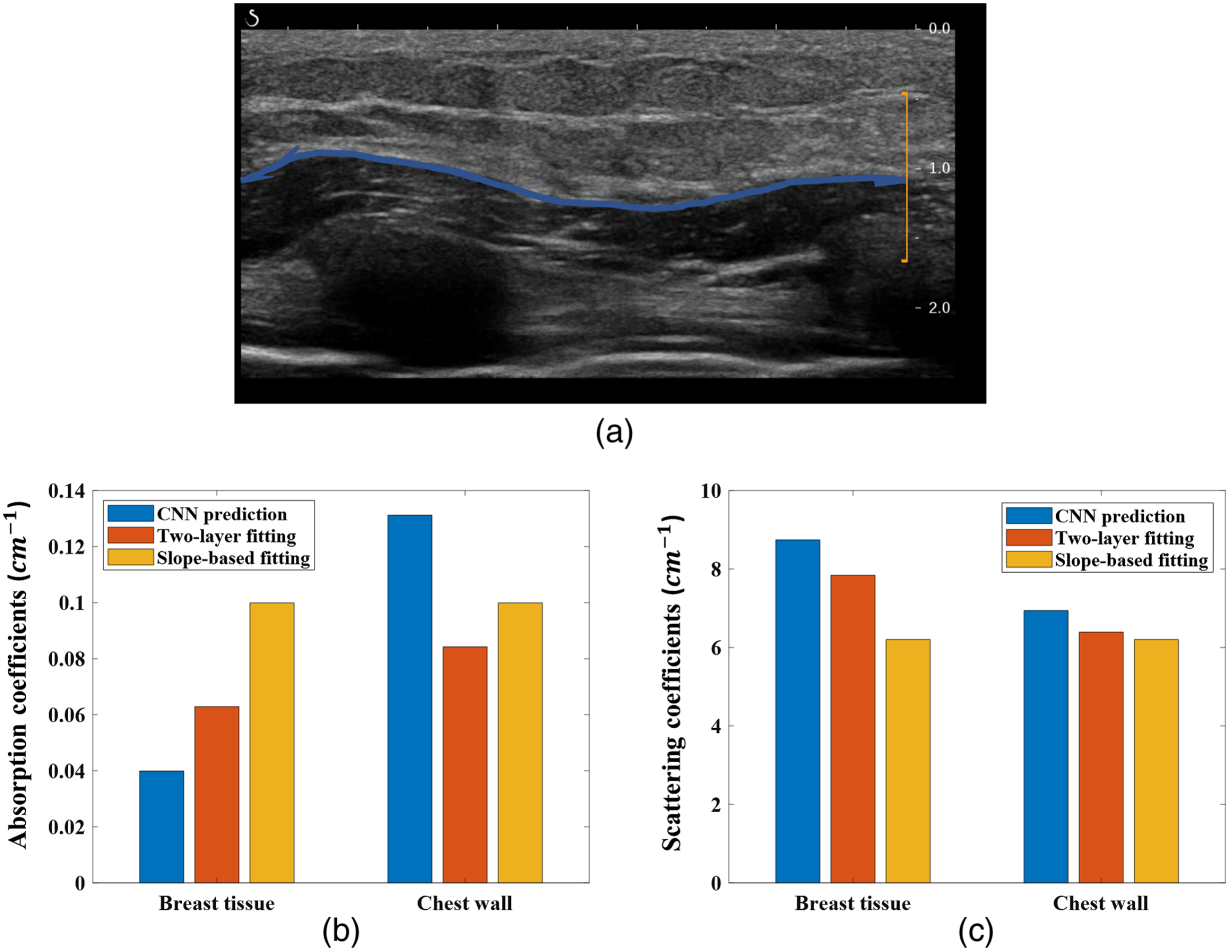
Co-registered US image and the predicted optical properties from the CNN model, and the fitted values from the two-layer fitting algorithm and the slope-based fitting algorithm, based on data acquired at 780 nm. (a) Co-registered US image, with the chest wall marked by the blue line. (b), (c) Predicted and fitted optical properties for both the breast tissue and chest wall. Blue bars are the predicted values; red bars are the fitted values.

To test the CNN model on clinical data, 12 patients with breast lesions were studied. For each patient, 4 to 11 sets of optical measurements were collected on the healthy side breast, using our hand-held probe. One clinical example is shown in Fig. 9, where the chest wall is located around 1 cm deep and is marked on the co-registered US image with a blue line. The CNN predicted that $\mu_{a}$ of the breast tissue is $0.0399\;{\mathrm{cm}}^{-1}$, the two-layer algorithm fitted $\mu_{a}$ is $0.0629\;{\mathrm{cm}}^{-1}$, and the slope-based algorithm fitted $\mu_{a}$ is $0.0999\;{\mathrm{cm}}^{-1}$. Both the two-layer fitted value and the slope-based fitted values are significantly higher than the predicted value. The CNN-predicted $\mu_{a}$ is within the range of the documented breast tissue values in Refs. 35 and 36, but both the two-layer algorithm fitted value and the slope-based algorithm fitted $\mu_{a}$ are significantly higher than the documented values.

**Fig. 10:**
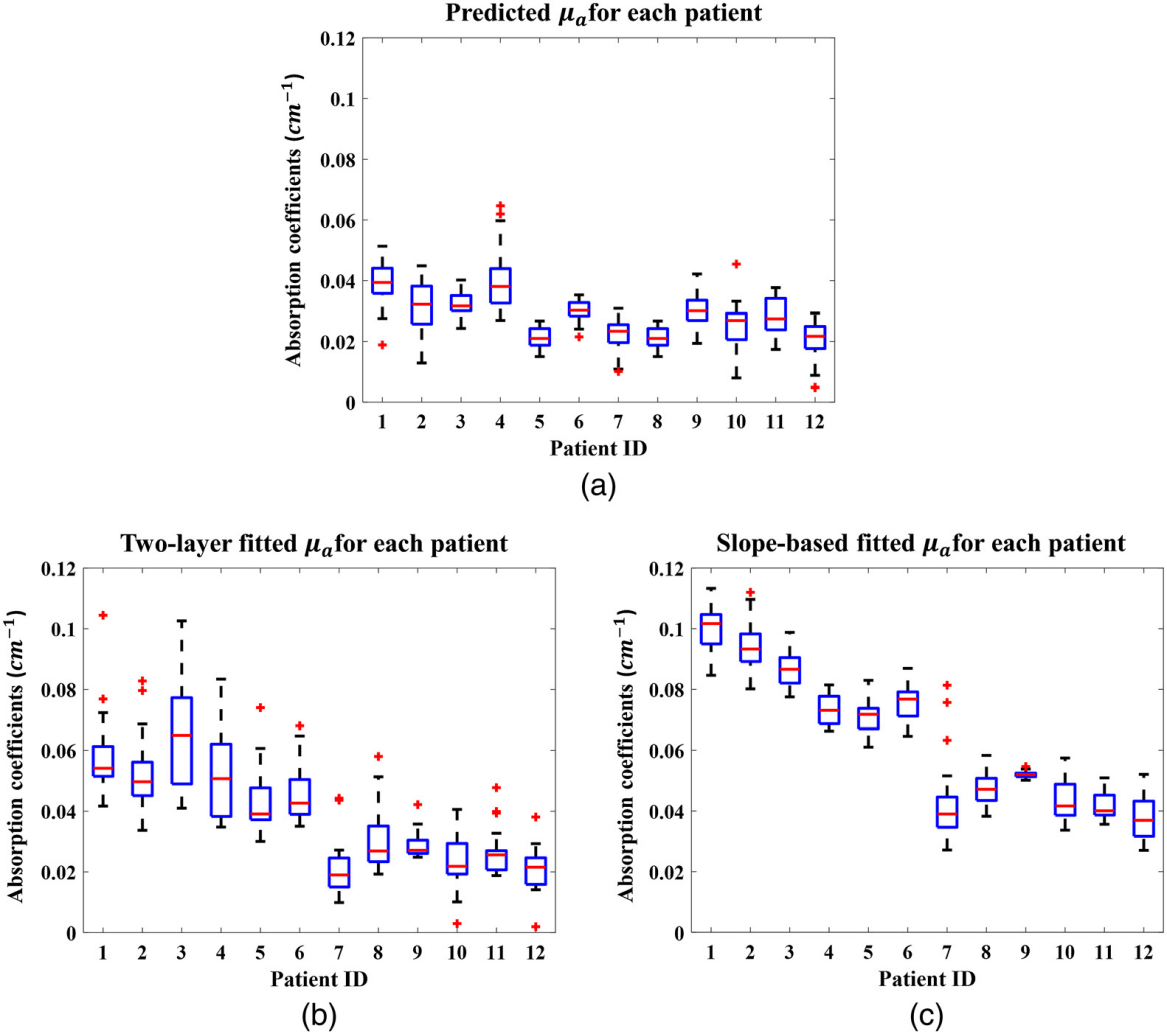
Boxplots of the predicted optical properties from the CNN model and the fitted values from the two-layer fitting algorithm and the slope-based fitting algorithm for all patients. The x-axis is patient IDs, arranged by chest wall depth, from shallowest to deepest.

Figure 10 shows the predicted $\mu_{a}$ and fitted $\mu_{a}$ values for all 12 patients, where it is apparent that the slope-based fitting always estimates higher values than the CNN. The predicted values from the CNN model vary from patient to patient, but show a low standard deviation within each patient, which indicates that the neural network model is robust for clinical data.

The x axis of Fig. 10, the patient ID, is ranged by the depth of the chest wall. Combined with Table 3, which contains each patient’s chest wall depth information, we can see that shallower chest walls lead to higher slope-based fitted values (c), but the predicted values are consistent, which agrees with the simulation and phantom results. The two-layer algorithm fitted value is relatively lower than the slope-based algorithm fitted value, but higher than the CNN predicted value. It also has a much higher standard deviation within each patient (b). The two-layer fitting algorithm is heavily dependent on the initial values, which are determined by the slope-based fitting algorithm. Hence, small variations in the slope-based fitted values can lead much larger variations in the two-layer fitted values. Other optical properties all follow the same trend and agree with the simulation and phantom results.

**Table 3:** Chest wall depths.

Patient ID (arbitrary)	1	2	3	4	5	6	7	8	9	10	11	12
Depth (cm)	1.2	1.3	1.5	1.8	1.8	2.0	2.0	2.0	2.0	2.2	2.3	2.5

## 4 Discussion and Conclusion

We presented a deep learning-based approach to accurately estimate the optical properties of both breast tissue and the chest wall, properties that are essential in constructing an accurate forward model for DOT reconstruction. A neural network model was trained on simulation data to learn the nonlinear mapping between normal tissue optical properties and reflectance measurements collected from the tissue surface, then fine-tuned with phantom data to further improve its performance. We validated the approach on simulation data, realistic digital breast phantoms, phantom experimental data, and clinical data. It successfully reduced the error in estimating the optical properties of both the breast tissue (to <10% of the ground truth values) and the chest wall (<20% of the ground truth values), and it is robust to different chest wall depths. Using Intel^®^ Core™ i5-6400 CPU, the algorithm takes around 20 s to predict the optical properties after the optical measurements have been inputted into the neural network. Most of the time is consumed in loading the model at first use.

Although the presented approach performed well on heterogeneous digital breast phantoms, these phantoms are nevertheless simple models, with only fat and fibroglandular tissues, whereas the human breast contains many more tissue types and more complex structures. With the presence of a smaller heterogeneity in the healthy breast, our data suggest that it will not significantly affect the CNN predicted average optical properties. For example, we have imbedded a lesion inside a digital breast phantom with 40% fat tissue ($\mu_{a}=0.02\;{\mathrm{cm}}^{-1}$ and $\mu_{s}^{\prime }=5\;{\mathrm{cm}}^{-1}$) and 60% glandular tissue ($\mu_{a}=0.04{\;\mathrm{cm}}^{-1}$ and $\mu_{s}^{\prime }=8\;{\mathrm{cm}}^{-1}$). The chest wall ($\mu_{a}=0.1\;{\mathrm{cm}}^{-1}$ and $\mu_{s}^{\prime }=7\;{\mathrm{cm}}^{-1}$) was placed 2 cm underneath the tissue surface. A small target of 1 cm in size was placed 1.5 cm underneath the tissue surface. We tested two optical contrasts of a lower $\mu_{a}$ of $0.1\;{\mathrm{cm}}^{-1}$ and a higher $\mu_{a}$ of $0.2\;{\mathrm{cm}}^{-1}$. The ground truth values for the non-target breast tissue were computed as the weighted average of the fat tissue and the glandular tissue, which were $\mu_{a}=0.032\;{\mathrm{cm}}^{-1}$ and $\mu_{s}^{\prime }=6.8\;{\mathrm{cm}}^{-1}$ in this case. With no target inside, the CNN predicted a $\mu_{a}$ of $0.0314\;{\mathrm{cm}}^{-1}$ which was close to the ground truth. With the low contrast target $\mu_{a}=0.1\;{\mathrm{cm}}^{-1}$ inside, the CNN predicted a $\mu_{a}$ of $0.0356\;{\mathrm{cm}}^{-1}$, which was slightly higher because of the target inside. With the high contrast target $\mu_{a}=0.2\;{\mathrm{cm}}^{-1}$ inside, the CNN predicted a similar $\mu_{a}$ of $0.0364\;{\mathrm{cm}}^{-1}$ as compared with the low contrast case. Thus, small heterogeneous has minimal effect on the estimated average optical properties.

The proposed approach can be further improved using more complex simulations and phantom experimental settings, such as heterogeneous breast tissue and complex tissue and chest wall interface. Also, in the clinical data, we do not have ground truth values for the optical properties of the breast tissue and the chest wall, so we cannot fine-tune the CNN model with the clinical data. However, the results of the predicted values and the fitted values of the clinical data agree with the results of simulation and phantom experiments, which is a promising indication that the CNN model can be directly applied to clinical studies.

The presented algorithm is developed for more accurate estimation of average optical properties of breast tissue using data from US-guided frequency-domain DOT measurements. However, it is directly applicable to diffuse optical spectroscopy (DOS) measurements when the underlying tissue has a layered structure and the depth of the first layer of tissue can be estimated by US or other methods. DOS has been widely used in measuring breast, brain, thyroid, and abdomen tissue optical properties.^37^^,^^42^^–^^44^

In summary, we have developed a deep learning-based neural network algorithm that more accurately estimates average breast tissue optical properties, improving the forward model for breast lesion image reconstruction.
